# *Lymphocyte Antigen 75* Polymorphisms Are Associated with Disease Susceptibility and Phenotype in Japanese Patients with Inflammatory Bowel Disease

**DOI:** 10.1155/2016/6485343

**Published:** 2016-11-14

**Authors:** Atsuhiro Hirayama, Satoru Joshita, Kei Kitahara, Kenji Mukawa, Tomoaki Suga, Takeji Umemura, Eiji Tanaka, Masao Ota

**Affiliations:** ^1^Department of Medicine, Division of Gastroenterology and Hepatology, Shinshu University School of Medicine, Matsumoto, Japan; ^2^Department of Gastroenterology, Japanese Red Cross Society Suwa Hospital, Suwa, Japan; ^3^Department of Legal Medicine, Shinshu University School of Medicine, Matsumoto, Japan

## Abstract

Recent genome-wide association studies have rapidly improved our understanding of the molecular pathways leading to inflammatory bowel disease (IBD), which includes Crohn's disease (CD) and ulcerative colitis (UC). Although several reports have demonstrated that gene single nucleotide polymorphisms (SNPs) are associated with susceptibility to IBD, its precise genetic factors have not been fully clarified. Here, we performed an association analysis between* lymphocyte antigen 75* (*LY75*) genetic variations and IBD susceptibility or phenotype. SNPs were genotyped in 51 CD patients, 94 UC patients, and 269 healthy controls of Japanese ethnicity. We detected a significant relationship with CD susceptibility for the rs16822581* LY75* SNP (*P* = 0.045). One haplotype (GT, *P* = 0.042) was also associated with CD susceptibility, while another carrying the opposite SNP (CA) was linked to an absence of surgical history for CD. Our findings confirm that* LY75* is involved in CD susceptibility and may play a role in disease activity in the Japanese population.

## 1. Introduction

Inflammatory bowel disease (IBD), which includes Crohn's disease (CD) and ulcerative colitis (UC), is a chronic destructive digestive disorder leading to tissue damage, loss of function, disability, and systemic inflammation [1]. CD is characterized by inflammation appearing in any region of the gastrointestinal tract and on the entire wall of the bowel, while UC is restricted to the mucosa of the colon. The number of patients with IBD has been increasing in both developed and developing countries to create clinical and economic problems. It is currently estimated that 1.4 million, 2.2 million, and 0.2 million individuals suffer from IBD in the United States, Europe, and Japan, respectively [2–4]. The precise etiology of this disease group remains poorly understood, although the loss of barrier function in the gut leading to an inappropriate inflammatory response to intestinal microbes [5] and reactivity of infiltrating T cells [6] in genetically predisposed individuals has provided insights into the pathogenesis of IBD [7].

Genome-wide association studies (GWAS) on European populations have uncovered several susceptibility genes to IBD [8] while meta-analyses have identified 71 CD susceptibility loci [9] and 47 UC susceptibility loci [10]. Genes implicated in the type 17 helper T-cell- (Th17-) interleukin-23 (IL-23) (Th17-IL23) pathway have been linked to both diseases and the etiology of IBD. Moreover, a meta-analysis of GWAS that analyzed more than 38,000 IBD cases identified an additional 163 susceptibility loci for IBD among European populations [11]. In the Japanese, several susceptibility loci for CD [12, 13] and UC [14] were discovered outside of the major histocompatibility complex (MHC) region by GWAS [15]. Based on these reports, multiple, but possibly similar, genes, including those for nucleotide oligomerization domain 2 [16] and the Th17-IL23 pathway, have been implicated in CD and UC onset despite ethnicity differences [17, 18]. Very recently, 38 genetic loci were associated with IBD via a trans-ancestry association study using genome-wide or Immunochip genotype data from an extended cohort of 86,640 European individuals and Immunochip data of 9,846 individuals sampled from ethnicities of East Asian, Indian, or Iranian descent [19], whereby one locus located in the vicinity of* lymphocyte antigen 75* (*LY75*) was reported to be associated with IBD susceptibility [19].

*LY75* encodes the endocytic receptor DEC-205, which is a member of the macrophage mannose receptor family of C-type lectins expressed at high levels by CD8^+^ dendritic cells (DCs) and thymic epithelial cells [20, 21]. CD8^+^ DCs expressing DEC-205 play a role in antigen processing and presentation in the context of both MHC class I and MHC class II molecules [20] and generate Th1 cell-mediated immune responses in an IL-12-independent, CD70-dependent mechanism [22, 23]. Therefore, the DEC-205 receptor is suspected to have an important role in T-cell function and homeostasis [24, 25].

As associations between* LY75* single nucleotide polymorphisms (SNPs) and susceptibility or phenotype have not been investigated in Japanese patients with IBD, this study examined such relationships in Japan.

## 2. Patients and Methods

### 2.1. Research Ethics Considerations

This study was conducted in accordance with the principles of the 1975 Declaration of Helsinki and approved by the ethics committees of both participating institutions (Shinshu University School of Medicine, Matsumoto, Japan: number 457, and Japanese Red Cross Society Suwa Hospital, Suwa, Japan: number 26-9). Informed written consent was obtained from all participants.

### 2.2. Subjects

We analyzed a total of 414 subjects (51 CD patients, 94 UC patients, and 269 healthy controls) recruited from Shinshu University Hospital in Matsumoto, Japan, and the Japanese Red Cross Society Suwa Hospital in Suwa, Japan. Subject information is summarized in Table 1. The participants had no direct relatives of non-Japanese ethnicity, and thus our cohort's racial background was considered to be uniformly Japanese. Control subjects were volunteers from hospital staff who had indicated the absence of any major illnesses and no direct familial relations in a standard questionnaire.

The diagnosis of CD or UC was confirmed by a combination of endoscopic, histopathological, radiological, and biochemical investigations according to existing guidelines [26, 27].

We also subdivided the IBD patients into disease parameters based on the phenotypes of disease location (ileal, colonic, ileocolonic, and not determined), history of intestinal resection, and presence of perianal disease for CD and disease location (proctitis, left-sided colitis, pancolitis, and not determined) for UC (Table 1).* Regarding extra-intestinal manifestations*,* only one patient with CD was complicated with polyarthritis and uveitis*,* while one patient with UC was complicated with polyarthritis*.

### 2.3. SNP Genotyping

Genomic DNA from patients was isolated by phenolic extraction of sodium dodecyl sulfate-lyzed and proteinase K-treated cells as described previously and adjusted to a concentration of 10–15 ng/*μ*L [28].

Five SNPs on the* LY75* gene (rs1365798, rs13307, rs11690117, rs16822581, and rs1511224) having minor allele frequencies of >5% were selected from HapMap Japanese data (https://www.ncbi.nlm.nih.gov/variation/tools/1000genomes/). All SNP genotyping was performed with a TaqMan 5′ exonuclease assay using primers supplied by Applied Biosystems (Foster City, CA, USA). The probe's fluorescence signals were detected with a StepOne Plus Real-Time PCR System (Applied Biosystems) according to the manufacturer's instructions.

### 2.4. Statistical Analysis

The R software Haploview version 4.2 [29] was used to evaluate the haplotype structure of the 5 SNPs on* LY75*. Pairwise linkage disequilibrium (LD) patterns and haplotype frequency analysis for all SNPs in patients and controls were analyzed by the block definition established by Gabriel et al. [30]. We assessed the significance of allele distribution between patients and controls using the *χ* ^2^ test by means of 2 × 2 comparisons (df = 1). A *P* value of less than 0.05 was considered to be statistically significant. We adjusted *P* values using Bonferroni's correction (*P*_*c*_) by multiplication of each locus by 5. Association strength was estimated by calculating the odds ratio (OR) and 95% confidence interval (CI). Statistical analysis of findings was performed using IBM SPSS Statistics version 21.0 (IBM, New York, NY, USA) and StatFlex version 6.0 (Artech Co., Ltd. Osaka, Japan) software.

## 3. Results

### 3.1. *LY75* Genotype Analysis of Patients with IBD and Controls

Five SNPs in the* LY75* gene (rs1511224, rs16822581, rs11690117, rs13307, and rs1365798) were genotyped in all IBD patients and controls. The genotype frequencies of the tested SNPs were in Hardy–Weinberg equilibrium in all subject groups (Table 2). No statistical differences were found in allele frequencies between patients with CD or UC and healthy controls (Table 3). However, the genotype frequency of rs16822581 in a recessive model exhibited a statistical difference between CD patients and controls after adjustment by Bonferroni's correction (*P* = 0.045) (Table 4). No such differences were detected for UC patients and controls (Table 4).

### 3.2. *LY75* Haplotype Analysis between Patients with IBD and Controls

**Figure 1:**
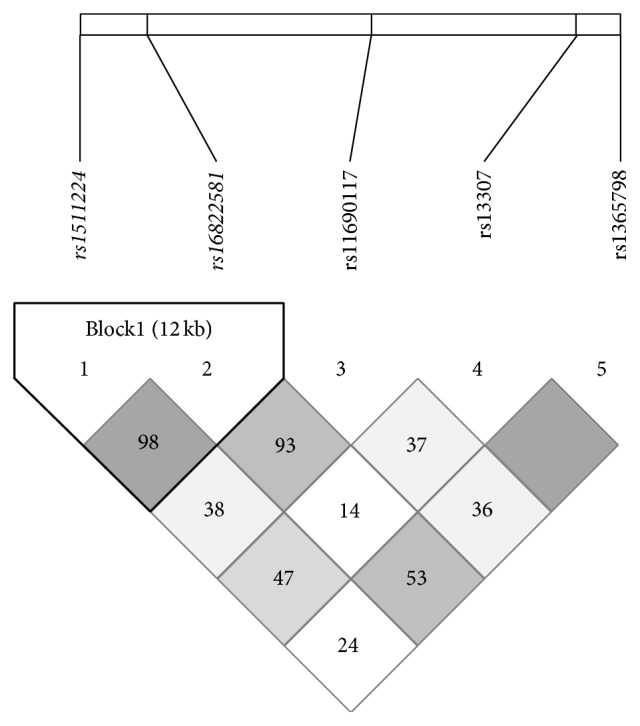
Linkage disequilibrium (LD) plot of 5 SNPs in* LY75* in 269 healthy subjects. Values of *r*2 corresponding to each SNP pair are expressed as a percentage and shown within the respective square.

To estimate haplotype frequency and analyze the associations between patients with IBD and healthy controls, tag SNPs were selected using the Tagger algorithm of the Haploview program. Two tag SNPs (rs1511224 and rs16822581) were found to be in strong LD among patients with CD or UC as well as in healthy controls (Figure 1). The top 3 haplotype frequencies in the blocks are shown in Table 5. Haplotype 2 (GT) was significantly associated with CD susceptibility (43% versus 33%; *P* = 0.042, OR 1.560, 95% CI 1.014–2.402) as compared with controls.

### 3.3. Associations between* LY75* SNPs, Haplotypes, and Clinical Features in Patients with CD

Among the patients with CD, 39% had received prior bowel resection and 43% had perianal disease (Table 1). However, the allele frequencies of the 5* LY75* SNPs showed no differences compared with controls for the presence or absence of surgical history, small intestine lesions, or perianal disease (Table 6).

Interestingly, haplotype 1 (AC) was significantly negatively correlated with a history of bowel resection in patients with CD (28% versus 48%; *P* = 0.036) (Table 7). No other associations were found for the presence or absence of small intestinal lesions or perianal disease.

## 4. Discussion

The present study investigated the associations between* LY75* SNPs and IBD susceptibility or phenotype in the Japanese population. Our key findings were as follows: (1) specific* LY75* polymorphisms were significantly associated with CD susceptibility; (2) there were no significant genetic associations between* LY75* SNPs and UC susceptibility; and (3) there was a relationship between a specific* LY75* haplotype and a history of bowel resection in patients with CD.

Previous studies have demonstrated that genetic polymorphisms in the MHC region strongly influenced both CD and UC, with several other SNPs located in non-MHC regions also associated with disease susceptibility. Moreover, genes in the epithelial barrier and the Th17-IL23R pathway have been related to CD and UC across multiple ethnicities. Since the DEC-205 receptor encoded by* LY75* has an important role in T-cell function and homeostasis in the epithelial barrier of the gut, we examined associations between* LY75* SNPs and disease susceptibility and uncovered a statistical difference for the rs16822581 SNP by a recessive model between CD patients and controls and for haplotype 2 (GT), which carried the disease susceptibility T allele at this SNP, indicating that* LY75* polymorphisms might be associated with CD predisposition in Japan as well. An earlier study demonstrated that a locus in* LY75* was linked to IBD onset [19]. Thus, our findings support shared genetic participation over ethnicities to be involved in IBD etiology, as documented for the Th17-IL23 pathway for CD and UC [17, 18].

A* LY75* locus variant has also been associated with erythema nodosum as an extra-intestinal manifestation of CD [31]. However, the lack of patients with this complication in our study made it impossible to conclude any relationship with the* LY75* SNPs. As the prevalence of erythema nodosum as an extra-intestinal symptom of CD is 2.3% [32], larger studies are needed to conclude if* LY75* SNPs are related to this complication in Japanese CD patients.

Interestingly, our study revealed 1 haplotype on the* LY75* gene to be associated with the disease phenotype of an absence of surgical history in patients with CD. Some patients required surgical resection due to tissue damage, loss of function, disability, or obstruction within the gut. This haplotype carried the opposite SNPs to disease susceptibility, thus confirming that* LY75* function was indeed associated with disease activity in CD.

The current study was preliminary in nature because the numbers of cases and controls were limited. To overcome type I error, larger studies are needed to validate the relationships between* LY75* gene polymorphisms and the expression and functions of their gene products.

## 5. Conclusions

Our findings confirm that* LY75* SNPs and haplotypes contribute to CD susceptibility and may play a role in disease activity. Further investigation is required to clarify precisely how* LY75* functions in the pathogenesis of CD.

## Figures and Tables

**Table 1:** Demographic and clinical data of CD, UC, and healthy subjects.

Characteristic	CD (*n* = 51)	UC (*n* = 96)	Controls (*n* = 269)
Age, years	44 (36–52)	49 (39–63)	40 (34–52)
Female/male	15/36	43/51	205/64
Disease phenotype			
Location^*∗*^	13/18/17/3	44/27/13/10	
Past surgical history	20 (39)		
Presence of anal lesion	22 (43)		

Data are expressed as number (%) except for age, expressed as median (first-third quartile).

^*∗*^Location was defined as ileal, colonic, ileocolonic, and not determined in CD and as proctitis, left-sided colitis pancolitis, and not determined in UC.

CD, Crohn's disease; UC, ulcerative colitis.

**Table 2:** Allele frequencies of SNPs in the *LY75* gene in CD, UC, and healthy subjects.

SNP number	dbSNP	Alleles (1>2)	Position (bp)	Gene location	CD (*n* = 51)		UC (*n* = 94)		Controls (*n* = 269)	
					MAF (%)	HWE *P* value	MAF (%)	HWE *P* value	MAF (%)	HWE *P* value
1	rs1511224	G>A	159894350	Intron	0.402	0.163	0.489	0.645	0.480	0.762
2	rs16822581	C>T	159882166	Exon	0.431	0.221	0.335	0.619	0.329	0.893
3	rs11690117	C>A	159841125	Intron	0.431	0.072	0.473	0.518	0.441	0.397
4	rs13307	C>T	159803485	Intron	0.402	1.000	0.346	1.000	0.357	0.922
5	rs1365798	C>T	159795853	3′ UTR	0.186	0.254	0.282	1.000	0.307	0.704

SNPs, single nucleotide polymorphisms; CD, Crohn's disease; UC, ulcerative colitis; MAF, minor allele frequency; HWE, Hardy–Weinberg equilibrium.

**Table 3:** Allele frequencies of 5 SNPs in CD, UC, and healthy controls.

SNP number	Allele	CD (*n* = 51)	*P*	*P*_*c*_	OR	95% CI	UC (*n* = 94)	*P*	*P*_*c*_	OR	95% CI	Controls (*n* = 269)
1	G	0.60	0.150	0.750	1.37	0.89–2.11	0.51	0.817	1.000	0.96	0.69–1.34	0.52
	A	0.40					0.49					0.48
2	C	0.57	0.046	0.230	1.55	1.01–2.38	0.67	0.878	1.000	1.03	0.72–1.46	0.67
	T	0.43					0.34					0.33
3	C	0.57	0.865	1.000	1.04	0.68–1.59	0.53	0.435	1.000	0.88	0.63–1.22	0.56
	A	0.43					0.47					0.44
4	C	0.60	0.386	1.000	1.21	0.79–1.87	0.65	0.784	1.000	0.95	0.67–1.35	0.64
	T	0.40					0.35					0.36
5	C	0.81	0.014	0.070	0.52	0.30–0.88	0.72	0.524	1.000	0.89	0.62–1.28	0.69
	T	0.19					0.28					0.31

SNPs, single nucleotide polymorphisms; CD, Crohn's disease; UC, ulcerative colitis; *P*_*c*_, corrected *P *value; OR, odds ratio; 95% CI, 95% confidence interval.

**Table 4:** Genotype distributions of *LY75* gene polymorphisms in CD, UC, and healthy subjects.

SNP number	Alleles (1>2)	Genotype	Genotype frequency, %			Model^*∗*^	Controls versus CD			Controls versus UC		
			CD	UC	Controls		*P*	*P*_*c*_	OR (95% CI)	*P*	*P*_*c*_	OR (95% CI)
			(*n* = 51)	(*n* = 94)	(*n* = 269)							
1	G>A	AA/AG/GG	21.6/37.3/41.2	25.5/46.8/27.7	22.3/51.3/26.4	Recessive	0.908	1.000	1.04 (0.50–2.16)	0.523	1.000	0.84 (0.49–1.44)
						(GG + AG versus AA)						
2	C>T	TT/TC/CC	23.5/39.2/37.3	12.8/41.5/45.7	10.4/45.0/44.6	Recessive	0.009	0.045	2.65 (1.24–5.64)	0.530	1.000	1.26 (0.61–2.59)
						(CC + TC versus TT)						
3	C>A	AA/AC/CC	25.5/35.3/39.2	24.5/45.7/29.8	20.8/46.5/32.7	Recessive	0.457	1.000	0.77 (0.38–1.54)	0.460	1.000	0.81 (0.47–1.41)
						(CC + AC versus AA)						
4	C>T	TT/TC/CC	15.7/49.0/35.3	11.7/45.7/42.6	13.0/45.4/41.6	Recessive	0.608	1.000	1.24 (0.54–2.86)	0.743	1.000	0.89 (0.43–1.82)
						(CC + TC versus TT)						
5	C>T	TT/TC/CC	0/37.3/62.7	7.4/41.5/51.1	10.0/41.3/48.7	Recessive	0.018	0.090	—	0.458	1.000	0.72 (0.30–1.72)
						(CC + TC versus TT)						

CD, Crohn's disease; UC, ulcerative colitis; SNP, single nucleotide polymorphism; *P*_*c*_, corrected *P *value; OR, odds ratio; 95% CI, 95% confidence interval.

^*∗*^The model with the smallest Akaike's information criterion value was defined as the best model for each SNP.

**Table 5:** *LY75* haplotypes in CD, UC, and healthy subjects.

Haplotype	SNP number		Frequency			CD versus controls			UC versus controls		
	1	2	CD (*n* = 51)	UC (*n* = 94)	Controls (*n* = 269)	*P*	OR	95% CI	*P*	OR	95% CI
1	A	C	0.40	0.48	0.48	0.160	0.74	0.48–1.13	0.881	1.03	0.74–1.43
2	G	T	0.43	0.33	0.33	0.042	1.56	1.01–2.40	0.947	1.01	0.71–1.44
3	G	C	0.17	0.18	0.19	0.529	0.84	0.48–1.47	0.708	0.92	0.60–1.41

CD, Crohn's disease; UC, ulcerative colitis; SNP, single nucleotide polymorphism; OR, odds ratio; 95% CI, 95% confidence interval; *P* values were calculated by the *χ* ^2^ test by means of 2 × 2 comparisons (df = 1).

**Table 6:** Allele frequencies of *LY75* SNPs and phenotypes in patients with CD.

SNP number	Allele	Past surgical history				Small intestine lesion				Presence of perianal lesion			
		+	−	*P*	*P*_*c*_	+	−	*P*	*P*_*c*_	+	−	*P*	*P*_*c*_
		(*n* = 20)	(*n* = 31)			(*n* = 31)	(*n* = 17)			(*n* = 22)	(*n* = 28)		
1	G	0.73	0.52	0.036	0.179	0.60	0.56	0.718	1.000	0.55	0.64	0.324	1.000
	A	0.28	0.48			0.40	0.44			0.46	0.36		
2	C	0.53	0.60	0.475	1.000	0.55	0.56	0.922	1.000	0.59	0.54	0.581	1.000
	T	0.48	0.40			0.45	0.44			0.41	0.46		
3	C	0.60	0.55	0.607	1.000	0.58	0.61	0.767	1.000	0.55	0.59	0.660	1.000
	A	0.40	0.45			0.42	0.39			0.46	0.41		
4	C	0.65	0.57	0.390	1.000	0.57	0.59	0.822	1.000	0.64	0.55	0.403	1.000
	T	0.35	0.44			0.44	0.41			0.36	0.45		
5	C	0.80	0.82	0.980	1.000	0.81	0.85	0.771	1.000	0.77	0.86	0.407	1.000
	T	0.20	0.18			0.19	0.15			0.23	0.14		

SNPs, single nucleotide polymorphisms; CD, Crohn's disease; *P*_*c*_, corrected *P* value.

**Table 7:** Comparison of *LY75* haplotype frequencies and phenotypes in patients with CD.

Haplotype	SNP number		Past surgical history			Small intestine lesion			Presence of perianal lesion		
	1	2	+ (*n* = 20)	− (*n* = 31)	*P*	+ (*n* = 31)	− (*n* = 17)	*P*	+ (*n* = 22)	− (*n* = 28)	*P*
1	A	C	0.28	0.48	0.036	0.40	0.44	0.718	0.46	0.36	0.324
2	G	T	0.48	0.40	0.475	0.45	0.44	0.922	0.41	0.46	0.581
3	G	C	0.25	0.11	0.070	0.15	0.12	0.706	0.14	0.18	0.568

CD, Crohn's disease; SNP, single nucleotide polymorphism.
